# Association between asthma and female sex hormones: Associação entre asma e hormônios sexuais femininos

**DOI:** 10.1590/1516-3180.2016.011827016

**Published:** 2017-01-05

**Authors:** Raquel Prudente de Carvalho Baldaçara, Ivaldo Silva

**Affiliations:** I MD. Assistant Professor, Medicine, Universidade Federal do Tocantins (UFT), Palmas (TO), Brazil.; II MD, PhD. Adjunct Professor, Gynecology, Universidade Federal do São Paulo (SP), Brazil.

**Keywords:** Asthma, Gonadal steroid hormones, Women, Contraceptives, oral, Cytokines

## Abstract

**CONTEXT AND OBJECTIVE:**

The relationship between sex hormones and asthma has been evaluated in several studies. The aim of this review article was to investigate the association between asthma and female sex hormones, under different conditions (premenstrual asthma, use of oral contraceptives, menopause, hormone replacement therapy and pregnancy).

**DESIGN AND SETTING:**

Narrative review of the medical literature, Universidade Federal do Tocantins (UFT) and Universidade Federal de São Paulo (Unifesp).

**METHODS:**

We searched the CAPES journal portal, a Brazilian platform that provides access to articles in the MEDLINE, PubMed, SciELO, and LILACS databases. The following keywords were used based on Medical Subject Headings: asthma, sex hormones, women and use of oral contraceptives.

**RESULTS:**

The associations between sex hormones and asthma remain obscure. In adults, asthma is more common in women than in men. In addition, mortality due to asthma is significantly higher among females. The immune system is influenced by sex hormones: either because progesterone stimulates progesterone-induced blocking factor and Th2 cytokines or because contraceptives derived from progesterone and estrogen stimulate the transcription factor GATA-3.

**CONCLUSIONS:**

The associations between asthma and female sex hormones remain obscure. We speculate that estrogen fluctuations are responsible for asthma exacerbations that occur in women. Because of the anti-inflammatory action of estrogen, it decreases TNF-α production, interferon-γ expression and NK cell activity. We suggest that further studies that highlight the underlying physiopathological mechanisms contributing towards these interactions should be conducted.

## RESUMO

**CONTEXTO E OBJETIVO:**

A relação entre os hormônios sexuais e a asma tem sido investigada em diversos estudos. Esta revisão tem como objetivo descrever a relação entre hormônios sexuais (endógenos e exógenos) e a inflamação nas vias aéreas, especialmente na asma, em eventos diferentes (na asma pré-menstrual, durante o uso de anticoncepcionais, na menopausa, no uso de terapia hormonal e na gestação).

**TIPO DE ESTUDO E LOCAL:**

Revisão narrativa da literatura médica, Universidade Federal do Tocantins (UFT) e Universidade Federal de São Paulo (Unifesp).

**MÉTODO:**

Pesquisamos o Portal de Periódicos Capes, uma plataforma brasileira que fornece acesso a artigos nas bases de dados MEDLINE, PubMed, SciELO e LILACS. Os descritores utilizados foram asma, hormônios sexuais, mulheres e uso de anticoncepcionais, com base no "Medical Subject Headings".

**RESULTADOS:**

As associações entre hormônios sexuais e asma ainda permanecem obscuras. Em adultos, a asma é mais frequente em mulheres do que em homens. Além disso, a mortalidade por asma é significativamente maior no sexo feminino, destacando-se que o sistema imunológico sofre influência de hormônios sexuais, seja porque a progesterona estimula o fator bloqueador induzido pela progesterona e citocinas Th2 ou porque contraceptivos derivados de progesterona e estrógeno estimulam o fator de transcrição GATA-3.

**CONCLUSÕES:**

A associação entre asma e hormônios sexuais femininos permanece obscura. Nós especulamos que as flutuações do estrogênio são responsáveis pelas exacerbações da asma que ocorrem nas mulheres. Devido à ação anti-inflamatória do estrogênio há redução da produção de TNF-α, da expressão do interferon-γ e da atividade das células NK. Sugerimos que sejam realizados novos estudos para esclarecer os mecanismos fisiopatológicos dessas interações.

## INTRODUCTION

Asthma is a heterogeneous process that displays considerable phenotypic variability and affects 300 million people globally.^1^^,^^2^ It is characterized by the presence of inflammation, hyperresponsiveness and reversible airway obstruction. It is considered to be a public health problem that affects 21% of the Brazilian population.^3^^,^^4^ In Brazil, the mortality rate due to asthma among women is 0.241 per 100,000 inhabitants, whereas among men, it is 0.193 per 100,000 inhabitants.^5^ Among adults, epidemiological studies have demonstrated that the prevalence of asthma is higher among females than among males.^6^^,^^7^^,^^8^

The relationship between sex hormones and asthma has been evaluated in several studies.^9^^,^^10^ Sex-related differences in the risk, incidence and pathogenesis of a variety of lung diseases exist in humans.^11^ Among children, the prevalence is higher in boys than in girls.^12^ Interestingly, after puberty, the frequency and severity of asthma increase among girls, such that it becomes more common among women by the age of 20 years.^13^^,^^14^ After the menopause, the difference in asthma prevalence between men and women decreases.^14^ Thus, in the United States, 65% of all deaths due to asthma occur among women.^11^

The current paradigm for the pathogenesis of asthma is directly related to gene-environment interaction. Production of Th2 cells (T helper 2) involves the 5q32 region, which is located on the long arm of chromosome 5, in a cluster of genes encoding IL-4 (interleukin 4), IL-5 (interleukin 5), IL-13 (interleukin 13) and IgE (immunoglobulin E) levels.^15^ The transcription factors that relate to increased Th2 cytokine levels include STAT-5 (signal transducer and activator transducing-5) and GATA-3 (a transcription factor that promotes differentiation of Th2 cells from naïve T lymphocytes). GATA-3 stimulates growth of Th2 cells and inhibits differentiation to Th1 (T helper 1).^16^^,^^17^ T lymphocytes are important effector cells in relation to asthma, and activation of Th2 cells is considered to be important, especially in cases of asthma relating to atopy. However, immune responses to Th1 lymphocyte activation may be responsible for epithelial changes and activation of airway smooth muscle. In addition, as the disease becomes chronic, it may cause activation of Th1 lymphocytes with increased TNF-α expression (tumor necrosis factor) and IFN-γ (interferon gamma). In non-atopic asthma, a neutrophil inflammatory process may occur.^18^

Tregs (regulatory T cells) reduce proliferation and decrease Th2 levels and hence the inflammatory process in asthma cases.^19^ Tregs are essential for induction and maintenance of tolerance against antigens.^20^ In asthmatic patients, Tregs become reduced in number and function.^20^ Recently, other T helper cells were discovered (Th9 and Th17), and these cells are related to the physiopathological process and worsening asthma.^21^ The role of IL-17 in asthma is often investigated in patients with non-IgE-mediated non-atopic asthma with a predominance of neutrophils, because Th17 cell levels correlate with disease severity.^22^

Sex hormones play an important role in respiratory health, and hormone fluctuations may be responsible for exacerbations of asthma in women. Hormone fluctuations occur cyclically in reproductive-age women. For four days after menstruation, follicle-stimulating hormone (FSH), luteinizing hormne (LH) and 17-β-estradiol levels are low. During the follicular phase of the menstrual cycle (days 12-16), progesterone levels remain low, while FSH, LH and 17-β-estradiol levels reach a peak. Finally, during the luteal phase (days 24-28 of the cycle), FSH and LH levels are low, whereas progesterone and 17-β-estradiol levels are moderately high.^23^ If pregnancy occurs, luteolysis is prevented and the progesterone and 17-β-estradiol levels remain high. After many years, as follicles are depleted and women reaches menopausal status, their sex hormone concentrations decrease to very low levels. In women using oral contraceptives, the progestin component suppresses secretion of LH, and the estrogenic component suppresses secretion of FSH, thus preventing ovulation.^12^

Asthmatic women need to be monitored for hormonal changes.^24^ In a study conducted by Scichilone that included eight healthy women, the progesterone levels during the menstrual cycle influenced the concentration of nitric oxide in exhaled air (FeNO) and alveolar exhaled nitric oxide (CANO).^25^ There is evidence suggesting that both endogenous and exogenous sex steroids modulate inflammatory processes in the lungs and in smooth muscle tissue during different phases of the hormonal cycle in women.^26^^,^^27^

A relationship between sex hormones and inflammatory responses in the lower airways, especially with regard to asthma, has been observed in several studies.^9^^,^^10^^,^^11^^,^^12^^,^^13^^,^^14^However, the mechanism for this interaction remains obscure. Thus, it is very important to review the main findings regarding interactions between sex hormones and to understand the pathophysiological mechanisms of this association.

## OBJECTIVE

To investigate the association between asthma and female sex hormones, at different conditions (premenstrual asthma, use of oral contraceptives, menopause, hormone replacement therapy and pregnancy).

## METHODS

For this narrative review, we searched for articles that addressed association between female sex hormones and asthma regardless of clinical situation, which could encompasse premenstrual period, pregnancy, post-menopause period, use of hormone replacement therapy or oral contraceptives. To do this, we searched the journals in the portal of the Coordination Office for Improvement of High-Education Personnel (Coordenação de Aperfeiçoamento de Pessoal de Nível Superior, CAPES). This is a Brazilian platform that provides access to bibliographic sources from various locations around the world, including the following: MEDLINE, PubMed, SciELO, and LILACS. The following keywords were used (based on Medical Subject Headings: https://www.nlm.nih.gov/mesh/): asthma and sex hormones (for the initial search); and women and oral contraceptives (included to refine the analysis). The inclusion criteria were the following: complete articles, published over the last 20 years and written in English or French. The exclusion criteria were the following: items for which the full content was not available, letters to the editor, editorials and articles published in non-scientific journals.

The search was performed in four steps:

1. Keywords search.
2. Preliminary search to include and exclude articles by using their abstracts.
3. Complete articles were read and additional exclusions were made.
4. Synthesis.

## RESULTS

### Results from search

**Figure 1:**
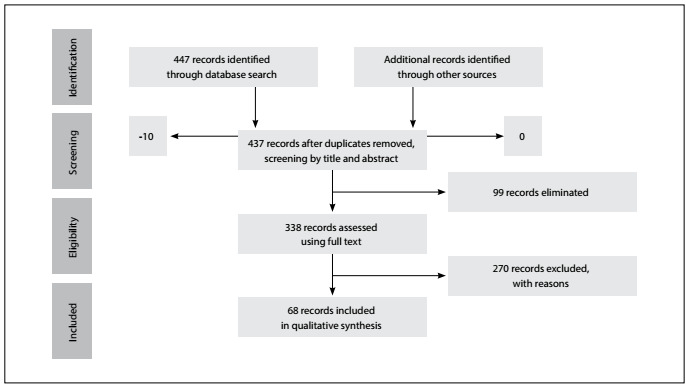
Flow diagram showing study selection for review of studies on association between asthma and female sex hormones.

In the initial search, we identified 447 references. However, through the preliminary analysis, only 68 references were selected. Only 16 were original articles. The process of study selection is presented in a flow diagram (Figure 1).

### Results from studies included

### Menstrual cycle and asthma

There is little data about airway physiology and hormonal fluctuations.^28^ Exacerbation of asthma in the form of premenstrual asthma (PMA) affects 30% to 40% of women with asthma.^29^^,^^30^ PMA was described for the first time by Frank in 1931, who reported on a woman who experienced severe attacks of asthma that occurred before her menstrual period.^31^ Some studies have reported a decrease in pulmonary function during the premenstrual portion of the cycle, with a decreased peak expiratory flow rate.^24^ There is also evidence for increased airway inflammation in patients with PMA, as demonstrated by increased levels of eosinophils in sputum and increased levels of fractionated exhaled nitric oxide.^32^

Tan et al. reported on abnormal regulation of beta2-adrenoreceptors, which was proposed as a possible mechanism for PMA during the period of the cycle when progesterone levels are high.^33^ The peak incidence of PMA complaints is two to three days before the onset of menstruation, but this phenomenon can also occur during both the menstrual and premenstrual intervals.^31^ In a prospective study on 182 female patients with asthma, 46% of all admissions to emergency departments due to acute asthma occurred during the perimenstrual period.^29^^,^^34^ Murphy reported that use of oral contraceptives was not protective, and further investigation was required to determine the mechanisms involved in PMA.^35^

A few studies have described treatments for PMA, with conflicting results. Several small series have described use of leukotriene receptor antagonists, exogenous intramuscular progesterone, xanthines,^14^^,^^24^ increased doses of inhaled corticosteroids, addition of long-acting beta2 agonists during the second half of the menstrual cycle, oral contraceptives, a single dose of estradiol (2 mg) during the luteal phase and gonadotropin-releasing-hormone (GnRH) analogues.^29^ However, more studies are needed in order to determine the appropriate treatment for PMA.

#### Use of hormone contraceptives and asthma

Contraceptives have frequently been used over the last 50 years for indications including hirsutism, irregular menstruation, dysmenorrhea, polycystic ovarian disease and contraception. Recently, clinical evidence has suggested that use of contraceptives is associated with impaired lung function.^7^^,^^36^ Some studies have suggested that use of contraceptives is a risk factor for development or exacerbation of asthma crises.^7^^,^^36^ The association between asthma and use of combined contraceptives (estrogen and progesterone) is unclear. The findings in the literature are divergent, given that some studies have reported that estrogen and progesterone improve total lung capacity and reduce the exacerbation of asthma symptoms, such as coughing, wheezing and dyspnea.^37^^,^^38^^,^^39^^,^^40^ In a study by Carlson et al., use of oral contraceptives (combined contraceptives) and unopposed forms of estrogens reduced hormone fluctuations and decreased premenstrual asthma.^41^ In a study by Lange, no relationship was found between use of oral contraceptives and asthma.^42^

Erkoçoğlu et al.^45^ found in a survey on 487 women by means of a questionnaire that 196 (40.2%) reported using oral contraceptives. This use was associated with higher risk of current wheezing among adolescents and young adults, but only among those who had taken the oral contraceptives recently during the previous year. In a study by Macsali et al.,^7^ women taking oral contraceptives had more asthma and allergies, but this association was not present in lean women, and there was an additional association with body mass index (BMI).

The association between asthma, obesity and sex hormones has been discussed in the medical literature. Obesity has been correlated with higher estrogen levels and with the enzyme aromatase, which in adipose tissue can convert androgens into estrogens.^43^^,^^44^ The Tucson Children's Respiratory Study showed a significant positive association between obesity and wheezing among women who reached puberty when they were under 11 years of age, while obesity was not associated with wheezing among women in whom puberty occurred after they were 11 years old. In the study by Erkoçoğlu et al., there was no evidence of a relationship between BMI and current wheezing.^45^ In a study by Nwaru and Sheikh, hormonal contraceptives reduced exacerbation of asthma and the number of episodes requiring care. That study also showed that overweight and obese women who do not use contraceptives may be at higher risk of asthma.^38^ In a study by Dratva et al., oral contraceptives also appeared to have a protective effect, through decreasing bronchial hyperreactivity.^39^

The cohort study by Jenkins et al. was the first to report an association between parity, use of oral contraceptives and the onset of asthma among women. In this study, women without asthma or wheezing by the age of seven years showed a lower risk of developing asthma, and the risk decreased by 7% per year of oral contraceptive pill use, independent of parity history. In this group (women without previous asthma or wheezing), the risk of current asthma increased for each birth (odds ratio, OR: 1.50; CI: 1.03-2.23; P = 0.04). Moreover, in the same group, the risk of current asthma was greater among women who were parous, according to the number of births. Women with one birth were at lower risk than nulliparous women. Among women who did have asthma or wheezy breathing by the age of seven years, neither reproductive history nor oral contraceptive pill use predicted current asthma.^46^

Some authors have suggested mechanisms to explain the complex interaction between hormonal contraceptives and asthma. Velez-Ortega reported on the impact of oral contraceptives on generation of induced regulatory T cells (iTregs).^37^ Dysregulation of iTregs plays a major role in the pathophysiology of asthma. In this study, patients taking oral contraceptives showed reduced serum sex hormone levels, and this was associated with higher rates of iTreg induction, better asthma control test scores and a tendency towards lower exhaled nitric oxide (eNO) levels.^37^ On the other hand, Guthikonda et al.^47^ reported that oral contraceptives and early menarche (via exogenous or endogenous hormones) were associated with the DNA methylation level of the Th2 transcription factor gene and GATA-3 and that they increased the risk of asthma among girls, possibly through interaction with genetic variants. This factor may explain how endogenous and exogenous hormones can, in women, increase the prevalence of asthma after puberty.^47^

Another mechanism was reported by Tan et al., who proposed that exogenous progesterone but not estradiol induces paradoxical downregulation and desensitization of β_2_-adrenoceptors in asthmatic women, compared with non-asthmatic subjects.^48^^,^^49^ Moreover, in another study on eleven women with stable mild to moderate asthma, Tan et al. reported that oral contraceptives did not alter β_2_-adrenoceptor regulation and function in stable female asthmatic patients.^33^

Finally, Salam et al.^26^ linked oral contraceptive use and asthma, both of which are common in young women. The outcomes from their study demonstrated that among women without asthma, oral contraceptive use was associated with higher risk of current wheezing. In contrast, in the same study, oral contraceptive use was associated with reduced prevalence of current wheezing among women with asthma. This paradox between hormonal contraceptives and immunologically unclear characteristics of sex hormones emphasizes the need for further research and the importance of knowing a patient's medical history, including the gynecological and hormonal characteristics of asthmatic women.^26^

In Table 1, we have summarized the differences between the results from different studies on asthma and hormone contraceptives. In Table 2, we have reported the main outcomes from animal model studies on sex hormones and asthma.

**Table 1:** Animal models for sex hormones and airway inflammation

Authors	Method	Results and conclusions
Hellings et al.^63^	BALB/c male mice of 6 weeks of age were sensitized to ovalbumin (Ova) using intraperitoneal injections. Medroxyprogesterone or placebo was instilled daily into the esophagus before and during the inhalatory Ova challenge phase.	Progesterone worsened allergic airway inflammation in Ova-challenged mice. Progesterone increased IL-5 levels and elevated airway eosinophilia. Progesterone did not influence allergen-specific IgE production. Progesterone aggravates the phenotype of eosinophilic airway inflammation in mice by enhancing systemic IL-5 production.
Degano et al.^64^	Ovariectomized seven-week-old female Wistar rats received either placebo or 17β-estradiol (E2) (10 to 100 mcg/kg/day) for 21 days. They were administered an aerosol of saline and increasing concentrations of acetylcholine (Ach) until lung resistance was observed.	Rats treated with low-dose E2 were less responsive to Ach than rats given either placebo or high-dose E2 were. Treatment with E2 had a differential, dose-dependent effect on airway responsiveness to Ach.
de Oliveira et al.^65^	The authors evaluated the roles of estradiol and progesterone in allergic lung inflammation. Female Wistar rats were ovariectomized (Ovx) and then sensitized with ovalbumin (OA). They received estradiol and progesterone.	In Ovx-allergic rats, treatment with estradiol decreased the amount of IL-10 and increased the amount of IL-4 produced by bone marrow (BM) cells. Estradiol increased IL1β and TNFα levels in BAL (bronchoalveolar lavage) cells. Progesterone increased the release of IL-10, IL-1β and TNFα by BAL cells and increased the production of IL-4 by BM cells. The existence of such dual hormonal effects suggests that hormone therapy in asthmatic postmenopausal women and women who suffer from premenstrual asthma should take into account the possibility that these treatments may worsen pulmonary conditions.
Mitchell et al.^66^	Adult female BALB/c mice were ovariectomized and implanted with time-release progesterone pallets. They were housed in filtered air or ETS (environmental tobacco smoke) for 6 weeks and exposed to HDMA (house dust mite allergen) by inhalation.	Progesterone alone did not increase mucous cell mass or abundance of eosinophils, but ETS coupled with progesterone exposure resulted in a significant increase in mucous cell metaplasia and increased accumulation of eosinophils in the asthma model. Progesterone, in the absence of estrogen, exacerbated the airway inflammation and airway remodeling that was induced by the toxicant ETS.
Matsubara et al.^67^	The authors compared sex differences in the development of airway hyperresponsiveness (AHR) following allergen exposure exclusively via the airways. Ovalbumin was administered via nebulization on 10 consecutive days in 8 to 10-week male and female BALB/c mice. After methacholine challenge, signiﬁcant AHR developed in male mice but not in female mice. Ovariectomized female mice showed signiﬁcant AHR after 10 days of Ova inhalation. ICI182,780, an estrogen antagonist, similarly enhanced airway responsiveness even when administered 1 hour before the assay.	The results showed that 17 beta-estradiol dose-dependently suppressed AHR in male mice. In all cases, airway responsiveness was inhibited by administration of a neurokinin 1 receptor antagonist. The neurokinin 1 receptor antagonist attenuated the effect that the estrogen receptor antagonist had in enhancing AHR in female mice *in vivo*. Endogenous estrogen may regulate the neurokinin 1-dependent prejunctional activation of airway smooth muscles in allergen-exposed mice.

**Table 2:** Hormone contraceptives and asthma

Authors and type of study	Method	Results and conclusions
Macsali et al.^7^ Cross-sectional survey	Postal questionnaires were sent to subjects in Denmark, Estonia, Iceland, Norway and Sweden from 1999 to 2001 (response rate in women, 77%). The analyses included 5791 women who were 25 to 44 years old, of whom 961 (17%) used oral contraceptives.	Oral contraceptive pills were associated with an increased risk of asthma, asthma with hay fever, wheezing and shortness of breath, hay fever and ≥ 3 asthma symptoms. Associations were present. Women using oral contraceptive pills had more asthma. This was found only in the normal weight and overweight women and not in lean women, thus indicating an interplay between sex hormones and metabolic status in their effects on airways.
Guthikonda et al.^47^ Cohort	Blood samples were collected from 245 female participants aged 18 years old.	Subjects with genotype AG showed an increase in the average risk ratio (RR) from 0.31 (95% CI: 0.10 to 0.8) to 11.65 (95% CI: 1.71 to 79.5) when the methylation level increased from 0.02 to 0.12 relative to the risk in genotype AA. A two-stage model that takes into account genetic variants of the GATA-3 gene, oral contraceptive use, age at menarche and DNA-methylation may explain how sex hormones can increase the prevalence of asthma after puberty.
Erkoçoğlu et al.^45^ Cross-sectional	The ISAAC questionnaire was provided to 487 women between 11.3 and 25.6 years of age. Questions on oral contraceptives were also asked.	In this study, n = 487 (ages ranged from 11.3 to 25.6 years old), 196 (40%) reported using an oral contraceptive, 7.4% had a diagnosis of asthma from a physician and 10.3% of them were active smokers. Young women taking oral contraceptives had a higher rate of current wheezing, thus suggesting that sex steroids may be important for respiratory health.
Dratva et al.^39^ SPALDIA 2 Cohort	571 women aged 28 to 58 years who had menstrual periods without hormone treatment were subjected to methacholine challenge. In a second step, 130 women taking oral contraceptives were subjected to methacholine challenge.	An effect of modification according to asthma status and oral contraceptive use was found, with a lower odds ratio (OR) among subjects without asthma. An OR < 1 was found among woman taking oral contraceptives. Oral contraceptives appeared to have a protective effect through which they decreased bronchial hyperreactivity.
Vélez-Ortega et al.^37^ Cohort	Thirteen patients were included in this pilot study. During three distinct phases of their menstrual cycles, the authors measured exhaled nitric oxide (eNO) levels, forced expiratory volume at 1 second (FEV_1_), asthma control test (ACT) scores, sex steroid hormone levels in serum, natural Tregs levels in peripheral blood, and the ability of CD4^+^ T cells to generate iTregs *ex vivo*.	Patients taking oral contraceptives showed reduced serum sex hormone levels in association with higher levels of iTreg induction, better ACT scores and a tendency to have lower eNO levels. The impact of sex hormones on the capacity of T cells to polarize towards a regulatory phenotype suggests that regulation of peripheral T cell lineage plasticity is a potential mechanism that may underlie the beneficial effects of oral contraceptives among women with asthma.
Tan et al.^33^ Cohort with intragroup analysis	The study population comprised 11 women aged 19 to 40 years with stable and moderate asthma. The patients were evaluated while on (day 20 to 21) and off (day 5 to 7) oral contraceptives during a 28-day calendar period.	Baseline FEV_1_ did not differ between patients who were on and off oral contraceptives. These did not alter beta2-adrenoreceptor regulation or function in stable female asthmatic patients.
Tan et al.^48^ Trial	Seven nonsmoking females aged 26 years with mild asthma completed the study. They were evaluated through two successive menstrual cycles during the follicular phase (days 1 to 6). They were randomized to receive single oral doses of either ethinyl estradiol or medroxyprogesterone.	The results showed that exogenous progesterone, but not estrogen, when given during the follicular phase, decreased beta2- adrenoreceptor density and cyclic-adenosine monophosphate (AMP) responses in female asthmatics. The beta2-adrenoreceptor was abnormally regulated in female asthmatics, and this might be a potential mechanism through which premenstrual asthma could be triggered when progesterone levels are high.
Salam et al.^26^ Cohort	905 women who had undergone menarche were included. The subjects ranged in age from 13 to 28 years and had participated in the Children's Health Study.	In women without asthma, oral contraceptive use was associated with higher risk of current wheezing. In contrast, oral contraceptive use was associated with reduced prevalence of current wheezing in women with asthma. These associations showed significant trends with duration of oral contraceptive use. Age at menarche was associated with new-onset asthma after puberty. Compared with women who had their menarche after they were 12 years old, women who reached their menarche before they were 12 years old were at higher risk of asthma after puberty. Because women have a higher risk of asthma after puberty, and because oral contraceptive use is common among young women, clinicians should inform women with asthma about the potential effects of oral contraceptives on asthma-related respiratory symptoms.
Jenkins et al.^46^ Cohort	681 women aged 29-32 years were randomly sampled from participants who were first surveyed at the age of 7 years in the 1968 Tasmanian Asthma Survey, which was a study of all children born in 1961 who attended school. Current asthma was defined as reporting asthma or wheezy breathing during the past 12 months.	The risk of current asthma in individuals who were parous increased with the number of births, while women with one birth were at lower risk than nulliparous women. Independent of parity, the risk decreased by 7% per year of oral contraceptive pill use. In women who had asthma or wheezy breathing by the age of 7 years old, neither reproductive history nor oral contraceptive pill use predicted current asthma. Parity and decreased oral contraceptive use predicted asthma in women, and these results are consistent with the hypothesis that the asthma that develops after childhood is in part a response to endogenous and exogenous female hormones.
Nwaru and Sheikh^38^ Cross-sectional survey	A population-based analysis using serial data from the Scottish general population. A total of 3257 non- pregnant, 16-45-year-old women were included.	The use of any hormonal contraceptive was associated with a reduced risk of current physician-diagnosed asthma. The use of a hormonal contraceptive may reduce asthma exacerbations. Overweight and obese non-contraceptive-using women may be at increasing risk of asthma.
Lange et al.^42^ Cross-sectional	Data from a study on women who were selected from the general population were used to correlate the effect of treatment with oral contraceptives and hormonal replacement therapy (HRT) with asthma indications. 377 women were on oral contraceptives (24.5% of the premenopausal women) and 458 were on HRT (15.2% of the postmenopausal women). The age span of the premenopausal women was 21-49 years and of the postmenopausal women, 27-90 years.	A weak association was observed between HRT and self-reported asthma. No relationship was found between the use of oral contraceptives and asthma, although an association was observed between asthma and HRT.

#### Postmenopausal hormone replacement therapy (HRT) and asthma

Among women over 50 years of age, the menopause can either coincide with the onset of asthma or be associated with deterioration of a pre-existing asthma condition.^50^ The definition of menopause is the cessation of menstruation for 12 months.^51^ The overall incidence of asthma decreases after the menopause,^14^ although in the Nurses' Health Study, use of hormone replacement therapy (HRT) approximately doubled the risk of asthma, compared with postmenopausal women without HRT. In that study, a 35% decrease in the incidence of asthma was observed among postmenopausal women without HRT.^10^ In a cohort study, Romieu et al. reported that the increase in the risk of asthma onset at the time of the menopause was only significant among women who reported using estrogen alone, especially among those who had never been smokers and those who had had an allergic disease before the onset of asthma. A small increase in the risk of asthma among women who used estrogen/progestogen was found in these subgroups.^52^ In a systematic review and meta-analysis, Zemp et al. found that there was no significant association between menopause with asthma prevalence or incidence except for women who reported using HRT.^53^

In a study by Carlson et al., HRT was associated with better lung function and an increase in forced expiratory volume at one second (FEV_1_).^41^ The mechanisms that link asthma and the menopause are unclear. After the menopause, FSH and LH levels are elevated, and estrogen levels decrease to the levels observed in patients with surgical oophorectomy, who also show extremely low progesterone levels. The incidence of asthma may be associated with decreased estrogen levels and a protective effect against the relative androgen excess that occurs during the menopausal transition.^53^^,^^54^ Clinical studies have indicated that the menopause is associated with exacerbation of pre-existing asthma. Thus, the onset of asthma is characterized by absence of atopy, absence of a family history and associations with urticaria and/or recurrent sinusitis of high severity.^23^ Balzano et al.^55^ showed that eosinophil levels were higher in the induced sputum of menopausal asthmatics, but Foschino Barbaro et al. reported that there were high sputum levels of neutrophils and exhaled interleukin (IL)-6 in women with menopausal asthma.^50^

Few studies have explored the link between the menopause and asthma. Hormonal processes and other factors, including genetics and inflammatory and metabolic characteristics, need to be taken into consideration. Studies have indicated that obesity has an effect on the severity of asthma and that this relationship is modified by gender. Estrogen and leptin levels (which have been correlated with increased airway inflammation in animal models)^56^ are higher in obese women than in non-obese women.^54^ Moreover, obesity has been shown to increase the risk of developing asthma. Interestingly, Gómez Real reported that lean women presented a higher risk of postmenopausal asthma than did obese women using HRT.^57^ This phenomenon can be explained by the notion that in lean women without insulin resistance, the pro-inflammatory effect of estrogens may predominate; while in obese women, the pro-inflammatory effects of estrogens are decreased through insulin resistance.^53^

#### Pregnancy and asthma

Asthma affects 3.7% to 8.4% of all pregnant women in the United States. Maternal asthma is associated with an increased risk of both maternal and fetal adverse perinatal outcomes,^58^ such that 20%-30% of women with asthma experience exacerbations that require medical intervention during pregnancy.^43^ There is also evidence of an increased risk of maternal mortality among some asthmatic women.^59^

A number of the physiological changes that occur during pregnancy can affect asthma status, including mechanical, immunological and hormonal alterations. Estradiol and progesterone levels are highest during pregnancy.^60^ Moreover, one third of women experience improved asthma, while another third of women retain the same asthma status and the remaining third experience worse asthma. Pregnancy is also marked by a state of Th2 dominance, and asthma is generally characterized by Th2 inflammation. Progesterone receptors are present in large quantities on the surface of lymphocytes, and binding of progesterone to its receptor induces stimulation and release of progesterone-induced blocking factor (PIBF) in a Th2 cytokine expression pattern (IL-4, IL-5, IL-6, IL-9, IL-10 and IL-13). The effects of these proteins are reduced in natural killer (NK) cells, in which expression of IFN-γ is decreased. NK cells are mainly observed in the endometrium of pregnant women.^12^^,^^61^ During the first trimester of pregnancy, the numbers of circulating and decidual regulatory T cells (Tregs) increase to promote tolerance at the maternal-fetal interface.^62^

Interestingly, fetal sex may influence asthma. Kwon et al. examined pregnant asthmatic women and found that carrying a female fetus was associated with worse maternal asthma than carrying a male fetus was.^60^ The mechanism that contributes towards this result is unclear, but there is evidence showing that testosterone potentiates the β-adrenergic-mediated relaxation of bronchial tissues and inhibits responses to histamine. Female sex is associated with higher maternal circulation of monocytes and upregulation of maternal inflammatory pathways.^58^

The mechanisms through which sex hormones influence asthma and the immunological characteristics of pregnancy at the maternal-fetal interface remain obscure, and new studies are needed in order to increase our understanding of and ability to manage asthmatic women.

## DISCUSSION 

Studies examining the role of hormonal factors in asthma among women have been conducted on human subjects and animal models, and the results have been described in reviews. In an attempt to understand the influence of sex hormones on pulmonary inflammatory responses, we discuss the main immunological aspects of sex hormones here.

Studies using animal models have demonstrated that both progesterone and estrogen can directly affect the lungs.^63^^,^^64^^,^^65^^,^^66^^,^^67^ Sex steroid hormones influence the immune system by acting on the structure and function of the thymus, thereby modulating the activity of B and T cells, mast cells and natural killer cells (NK cells), and affecting phagocytic cells and cytokine production. These hormones act via a variety of receptors (including the estrogen receptors ERα and ERβ; and the progesterone receptors PR-A and PR-B), and these steroid receptors have been described as nuclear receptors that act as transcription factors to regulate gene expression.^23^ However, it has been shown that some steroid receptors are located at the plasma membrane (e.g. membrane-bound G-protein-coupled receptors).^68^^,^^69^ These receptors are also expressed in the human lungs, such that sex hormones play a role in development of the lungs and androgen receptors are expressed in the mesenchymal and epithelial cells of the lungs.

Gender differences have been observed in relation to development of the lungs. For example, production of surfactants appears earlier in female than in male neonatal lungs, and male preterm infants are at higher risk of experiencing developmental distress syndrome. In addition, before puberty, the prevalence of asthma is higher among boys.^43^ Both male and female fetuses express androgen receptors (AR-A, AR-B) in non-reproductive tissues, with significantly higher numbers of AR-B than AR-A receptors expressed in the lungs. However, few studies have examined expression of androgens in inflammatory airways, and testosterone has been shown to cause relaxation of airway smooth muscles.^70^ Testosterone may increase apoptosis in T cells, thus resulting in a lower percentage of T lymphocytes in the total pool of lymphocytes in males than in females.^12^

In allergic asthma, airway inflammation is mainly characterized by Th2-mediated processes, including secretion of the cytokines IL-4, IL-5, IL-6, IL-9 and IL-13, secretion of chemokines, regulation of the activation of normal T cells (RANTES), and production of granulocyte macrophage colony-stimulating factor (GM-CSF). In patients with asthma and in allergic animal models (e.g. allergen-challenged mice), bronchoalveolar lavage contains large numbers of eosinophils, M2-polarized macrophages and activated mast cells. In several cases, the numbers of neutrophils in the bronchoalveolar lavage have been found to be higher as a result of Th17-mediated responses and production of IL-8.^68^^,^^69^ The airway epithelium in asthmatic patients recruits innate and adaptive cells via cytokines, including IL-25 and IL-33, and chemokines such as CCL2, CCL17 and CCL20, and it secretes transforming growth factor beta (TGFβ), which is responsible for airway remodelling.^69^

The transition of monocytes along the monocyte-macrophage axis is accompanied by upregulation of the 46 kDa ERα.^35^ Activated monocytes and macrophages show increased tumor necrosis factor-alpha (TNFα) secretion. TNFα is a cytokine produced by Th1 cells and is an important mediator in pro-inflammatory responses. Female reproductive phases also influence the production of TNFα by monocytes. In the luteal phase, higher plasma levels of TNFα have been observed.^12^ However, 17β estradiol may decrease TNFα levels via an anti-inflammatory effect caused by estrogen.^71^

Few studies have examined the effects of sex hormones on the bronchial epithelium. The human bronchial epithelium expresses both ERα and ERβ. In patients with asthma, estrogens facilitate dissociation of endothelial nitric oxide synthetase, which results in activation of the NO pathway, vasodilatation and increased inflammation.^72^ In another study, treatment of bronchial epithelial cells with 10 nM estrogen induced expression of NOS and production of nitric oxide, thus resulting in bronchodilation.^69^^,^^73^ In a study by Mandhane et al., among women who were not using oral contraceptives, an increase in progesterone level was associated with an increase in exhaled nitric oxide levels, thus indicating that an inflammatory process was associated with progesterone.^74^

Stimulation of Th2-mediated inflammatory responses and asthma by progesterone has been considered by many studies to represent a typical Th2 disorder.^69^^,^^75^ In a study by Loza et al., increased accumulation of IL-13^+^T cells (Th2) was observed in female but not in male asthmatics, and this association was maintained when the analysis was restricted to atopic subjects.^75^ In an animal model, ovariectomized or estradiol antagonist-treated mice developed reduced IL-5 dependent eosinophilia during allergic inflammation.^76^ However, depending on the concentration of estrogen, it may play dual pro and anti-inflammatory roles.^64^^,^^77^

## CONCLUSIONS

We have attempted to discuss the characteristics that are affected by sexual hormones during pulmonary inflammatory responses. However, the associations between these factors remain obscure. We speculate that estrogen fluctuations are responsible for asthma exacerbations that occur in women. Because of the anti-inflammatory action of estrogen, as this hormone decreases TNF-α production, it reduces IFN-γ expression, and NK cell activity. We suggest that further studies that highlight the underlying physiopathological mechanisms contributing towards these interactions should be conducted.
